# Adherence to ASCO for Prophylaxis of Acute Chemotherapy-Induced Nausea and Vomiting in Iran

**DOI:** 10.31557/APJCP.2020.21.6.1567

**Published:** 2020-06

**Authors:** Mahbobeh Ebrahimi, Valiollah Mehrzad, Azadeh Moghaddas

**Affiliations:** 1 *Department of Clinical Pharmacy, Faculty of Pharmacy, Isfahan University of Medical Sciences, Isfahan, Iran. *; 2 *Department of Oncology/Hematology, Faculty of Medicine, Isfahan University of Medical Sciences, Isfahan, Iran. *

**Keywords:** Chemotherapy, induced nausea and vomiting, American Society of Clinical Oncology, Guideline, Prophylaxis

## Abstract

**Introduction:**

Chemotherapy-induced nausea and vomiting (CINV) is one of the scariest chemotherapy-induced adverse effects. We evaluated the adherence to the 2017 American Society of Clinical Oncology (ASCO), the latest guideline recommendations, for the management of acute CINV at our institute.

**Methods:**

During a 6-months cross-sectional study on outpatient’s cancer patients, we collected data from the prescription documents during temporary hospitalization and compared the results with ASCO guideline recommendations.

**Results:**

The most prescribed prophylactic regimens for the management of CINV were combination of aprepitant, granisetron, and dexamethasone and metoclopramide (51.8%). Regarding prescription compatibility in our center with ASCO guideline recommnedations, selection of different regimens for prophylaxis of acute CINV in our institute was compliant in 0 %, 22%, 4%, and 40% of high, moderate, low, and minimal emetogenic potential of chemotherapy regimen groupss, respectively.

**Conclusion:**

Although our hospital is a referral and university-affiliated center, adherence to the ASCO guideline recommendations for prophylaxis of CINV was poor.

## Introduction

In spite of advances in the management and prevention of chemotherapy-induced adverse effects, chemotherapy-induced nausea and vomiting (CINV) remains as one of the scariest chemotherapy-induced adverse effects. Basically, up to 80% of patients receiving chemotherapy experience CINV, leading to complications, such as electrolyte imbalances, dehydration, malnutrition, and quality of life deterioration (Koth and Kolesar, 2017). 

Several medications, such as corticosteroids, serotonin and neurokinin antagonists, dopamine antagonists, and benzodiazepines have been used for the management of CINV. However, there is a significant concern regarding the toxicity, intolerance, and drug interaction of medications used for the prevention of CINV (Burmeister, et al., 2012). 

Optimum therapeutic standards have been applied in accordance with different evidence-based, updated, and practical medical guidelines. Those updated recommendations should be rapidly implemented in medical practice as they are released. The American Society of Clinical Oncology (ASCO) recommendations are the basis for medical oncology issues, such as CINV management, and are updated periodically according to scientific and new evidence (Hesketh et al., 2017). 

Cabana et al., (1999) found a variety of adherence barriers against applying guideline recommendations, such as physician unawareness, disagreement with the guidance, lack of belief in the effectiveness of guideline-driven management, and a propensity of being adhered to previous practice. 

There is a paucity of data on the adherence to guidelines for prophylaxis of CINV in clinical practice. Only a few studies addressed this issue, whose results were against the expectations, showing the difficulties in transferring clinical guideline recommendations into the practice (Lomas et al., 1989; Loprinzi et al., 2000; Mutnick and Szymusink-Mutnick, 1996). Therefore, this study was attempted to assess the adherence to the 2017 ASCO recommendations (Hesketh et al., 2017) for the management of acute CINV at the outpatient oncology center of Omid hospital, Esfahan, Iran.

## Materials and Methods

This study was approved by the Research Ethics Committee of the Isfahan University of Medical Sciences, Isfahan, Iran. Data collection was carried out in the outpatient oncology center of Omid hospital, which is a tertiary hospital affiliated to Isfahan University of Medical Sciences. 

We prospectively identified all adult patients who received chemotherapy for the first time at our institute between September 1, 2017, and April 30, 2018. 

Patients who received concurrent medications with high emetogenic potential, such as glucocorticoids or progestogens, in addition to chemotherapy drugs were excluded. We also excluded patients who had documented brain metastasis, received concurrent radiotherapy, and those with serum electrolyte abnormality. 

The baseline characteristics of patients, including age, gender, past medical history, oncology diagnosis, the chemotherapy regimens (doses and route of administration), and therapeutic intent was considered. During temporary hospitalization, we extracted all the prophylactic orders for the management or prevention of acute CINV (within 24 h after administration of chemotherapy), including doses and schedules of whole antiemetic medications, from the written prescription documents. In this study, anticipatory CINV was not considered because only the first cycle of chemotherapy was intended. In addition, the severity of CINV was categorized by VAS score during 24 h of chemotherapy (categorized into three-time periods, including 0-2, 2-6, and 6-24 h after chemotherapy administration).

After data collection, prescription compatibility between medication prescribed for the management of CINV in our institute and the latest ASCO guideline recommendations (Hesketh et al., 2017) was determined for each cancer patient by comparing the type, dose, and route of administration for prescribed medications. In this study, we defined adherence failure as follows:

(a) if a recommended antiemetic regimen was not prescribed,

(b) if a recommended dose was not administrated, 

or (c) if a non-recommended antiemetic was used. 

According to the ASCO guideline recommendations (Hesketh et al., 2017), the potential risk of emetogenic for chemotherapy regimens is divided into four groups: high risk (>90% of patients suffer from nausea/vomiting without prophylaxis), moderate risk (30–90% of patients suffer from nausea/vomiting without prophylaxis), low risk (10–30% of patients suffer from nausea/vomiting without prophylaxis), and minimal risk (<10% of patients suffer from nausea/vomiting without prophylaxis). Several prophylactic medications for the prevention of each category have been proposed.. (See supplementary scheme).

*Statistical analyses*

We used the Statistical Package for Social Sciences (SPSS Inc., Chicago, IL, USA) (version 20) for data analysis. Categorical variables were expressed as a percentage. Continuous variables were reported as mean± standard deviation (SD). The Wilcoxon signed-rank test and McNemar’s test were used to determine the differences regarding occurrences of CINV in different time periods. The possible association between the presence and severity of CINV with patients’ age and gender was assessed by Spearman’s rank correlation. A 2-tailed p-value <0.05 was considered as statistically significant. 

## Results

During follow-up, 139 patients fulfilled the inclusion criteria. The baseline characteristics of patients have been shown in Table 1. The mean patients’ age was 50 ± 12.8 years, and 97 patients (69.8%) were female. Solid tumors were the primary diagnoses of 117 out of 139 patients (84.2%). One hundred thirty-four (96.4%) of included patients received chemotherapy as a curative intent. 

The episode of nausea and vomiting were considered only in 110 cancer patients due to lack of patients’ cooperation. Generally, among eligible patients, 45 (40%) patients did not reporte any signs of CINV after receiving prophylactic regimen. However, as shown in Table 1, 20% of patients encountered severe chemotherapy-induced nausea during 6-24 hours after chemotherapy administration. During follow-up, we detected at least one episode of vomiting in 23 (20.1%) patients. In addition, we found that the chemotherapy-induced vomiting episodes after 2 hours of chemotherapy administration were significantly more in comparison with two other time peiords (P< 0.001) (Table 1). During the same time span, nearly 28.2% of our patients experienced at least one episode of vomiting. The rate of CINV was statistically more among female than male (P<0.045). In addition, an inverse relationship between the age of patients and the episode of CINV occurrences was observed (P< 0.001). 

The emetogenic potential of chemotherapy regimens was high in 39 (28.1%) patients, moderate in 70 (50.35%) patients; low in 25 (18%), and minimal in 5 (3.6%) patients. In the category of high emetogenic potential of chemotherapy regimens, the combination of epirubicine or doxurubicine and cyclophosphamide were the most prescribed regimens, 15 (10.8%) and 12 (8.63%) respectively. In the category of moderate emetogenic, potential of chemotherapy regimens, the combination of paclitaxel and carboplatin was the most frequent prescribed regimen (8.63%). In the category of low emetogenic potential of chemotherapy regimens, docetaxel (5.75%) was the most repeated regimen. 

The most prescribed prophylactic regimens for the management of CINV in our institute was combination of aprepitant, granisetron, dexamethasone, and metoclopramide (51.8%) followed by combination of aprepitant, granisetron, and dexamethasone (23%). 

Regarding compatibility between medication prescribed for the management of CINV in our institute and ASCO guideline recomendations (Hesketh et al., 2017), selection of different regimens for the prophylaxis of acute CINV in our hospital was compliant in 0%, 22%, 4%, and 40% of high, moderate, low, and minimal emetogenic potential of chemotherapy regimens, respectively (Table 2). However, the prescribed dose of regimens for the management of CINV was entirely in accordance with ASCO guideline recommendations (Hesketh et al., 2017) . 

As depicted in Table 2, in the highly emetogenic potential group, the most two essential reasons for lack of adherence to the ASCO guideline recommendations (Hesketh et al., 2017) were lack of oral olanzapine prescription or prescription of metoclopramide, as a dopamine antagonism. 

In the moderate emetogenic potential group, the over-prescription of metoclopramide and/or aprepitant was the most frequent prescription fault. In the low emetogenic potential group, the inappropriate administration of metoclopramide and/or aprepitant and /or granisetron was the most frequent prescription fault, and finally the overuse of metoclopramide, aprepitant, and granisetron were reported as the most frequent prescription fault in minimal emetogenic potential group. 

**Table 1:** Demographic and Clinical Characteristics of Patients (n = 139)

Baseline characteristics	Patients (n [%])	*P*-value
	Mean±SD	
Gender		
Male	42 (30.21)	
Female	97 (69.78)	
Age (years)	50 ± 12.81	
Body Mass Index (kg/m^2^)	62.98± 11.72	
Past Medical History		
Diabetes	25 (17.98)	
Hypertension	36 (27.33)	
Chemotherapy intention		
Curative	134 (96.4)	
Palliative	5 (3.59)	
Oncology diagnosis		
Breast cancer	57 (41)	
Colorectal cancer	25 (17.98)	
Lymphoma	9 (6.47)	
Ovarian cancer	7 (5.03)	
Lung cancer	6 (4.3)	
Prostate cancer	5 (3.5)	
Gastric cancer	4 (2.84)	
Other causes	26 (18.7)	
*Episode of nausea chemotherapy regimen administration		
Rate (N:65)		
0-2 hours (female/male)	15 (24)/2 (3.07)	P< 0.001
2-6 hours (female/male)	38 (58.46)/11 (16.92)	
6-24 hours (female/male)	45 (69.23)/13 (20)	
Severity (N:65)		
0-2 hours (female/male)		
Low	9 (13.83)/2 (3.07)	
Moderate	4 (6.15)/0	
High	2 (3.07)/0	
2-6 hours (female/male)		
Low	18 (27.69)/3 (4.61)	P< 0.001
Moderate	16 (24.61)/6 (9.23)	
High	6 (9.23)/0	
6-24 hours (female/male)		6 (9.23)/0
Low	19 (29.23)/5 (7.69)	
Moderate	10 (15.38)/4 (6.15)	
High	16 (24.61)/4 (6.15)	
*Episode of vomiting after chemotherapy regimen administration		
Rate (N:23)		
0-2 hours (female/male)	0/0	
2-6 hours (female/male)	9 (39.13)/0	
6-24 hours (female/male)	19 (82.60)/3 (13.04)	

*The episode of nausea and vomiting were considered only in 110 cancer patients due to lack of cooperation.

**Table 2:** Prevalence of Guideline-Consistent Acute CINV Prophylaxis for Single-Day Chemotherapy, by Emetogenicity of Chemotherapy and the Main Cause of Inconsistency (n = 139)

Emetogenic potential group	N (%) of ptients with guideline inconsistency	Guideline consistency (%)
High	39 (28.05)	0%
Lack of olanzapine prescription	13 (9.35)	
Lack of olanzapine but extra metoclopramide prescription	26 (18.70)	
Moderate		22%
Extra aprepitant prescription	55 (39.56)	
Extra metoclopramide prescription	7 (5.03)	
Extra both aprepitant and metoclopramide prescription	13 (9.35)	
Low		4%
Extra granisetron prescription	24 (17.26)	
Extra both granisetron	3 (2.15)	
Metoclopramide prescription	8 (5.75)	
Extra all aprepitant, granisetron and metoclopramide prescription	13 (9.35)	
Minimal		40%
Extra dexametasone prescription	3 (2.15)	
Extra both granisetron	1 (0.71)	
Metoclopramide prescription	1 (0.71)	
Extra all aprepitant, granisetron and metoclopramide prescription	1 (0.71)	

## Discussion

Our study revealed that the adherence to the ASCO 2017 clinical recommendations (Hesketh et al., 2017) for prophylaxis of CINV at our institute was not optimal. The total amount of non-adherence in all aspects was more than 83%. The significant deviation from the 2017 ASCO clinical recommendations (Hesketh et al., 2017) was due to lack of olanzapine prescription especially in highly emetogenic potential group of chemotherapy regimens, which has been newly added to the 2017 ASCO recommendation (Hesketh et al., 2017), or an unnecessary use of dopamine antagonists withdrawn from recently published 2017 ASCO clinical recommendations (Koth and Kolesar, 2017).

Burmeister et al., (2012) assessed adherence to the European Society of Medical Oncology (ESMO)/Multinational Association of Supportive Care in Cancer (MASCC) recommendations for prophylaxis of CINV at their institute. They found that 39 of 54 patients with low emetogenic potential regimens had a serotonin antagonist, and 24 of 100 with moderately emetogenic potential regimens had a neurokinin antagonist. They concluded that prophylaxis of acute CINV was not adherent in 39% of their patients. Nevertheless, 71% of their patients treated with highly emetogenic potential regimens received the guideline-specified prescription (Burmeister et al., 2012). More consistent results was noted in another study which demonstrated substantial compliance with pre-chemotherapy prophylaxis recommendations and little overuse of established pre-chemotherapy drugs in highly emetogenic potential regimens (Aapro et al., 2012). 

Management of CINV has been profoundly affected by the development of new antiemetic drugs in the past two decades. Since the late 1990s, three professional oncology organizations—the MASCC/ESMO, the ASCO, and the National Comprehensive Cancer Network (NCCN) have created CINV management guidelines from latest and available clinical research data (Tageja and Groninger, 2016). 

Understanding and implementing guideline recommendations have been noted critically due to improvement in cancer treatment and its associated complications, such as CINV (Mertens et al., 2003).

A recently updated ASCO guideline, including a comprehensive literature review and recommendations from data obtained from forty-one systematic reviews on prevention of CINV, was published (Hesketh et al., 2017). Key updates in the last version include the addition of oral olanzapine to antiemetic regimens for adults who receive high emetogenic potential regimens or who experience breakthrough nausea and vomiting. 

Olanzapine, an atypical antipsychotic drug that blocks dopaminergic, serotonergic, adrenergic, and histamine receptors, has been evaluated in combination with 5-HT3 receptor antagonist and corticosteroid for both acute and delayed CINV prophylaxis (Rapoport, 2017). 

A recently published meta-analysis reviewed data from 10 randomized controlled trials (RCTs), including the Phase III trial, and evaluated the efficacy of olanzapine and other antiemetic medications in the prevention of CINV (Olver et al., 2011). In the acute phase, authors found a significant difference in favor of olanzapine for the endpoint “no emesis” (relative risk [RR], 1.10; 95% confidence interval [CI], 1.03–1.17), but olanzapine was not superior concerning the second endpoint, “no nausea.” In the delayed phase, olanzapine had demonstrated superiority over comparator agents or regimens in terms of both the no-emesis (RR, 1.31; 95% CI, 1.14–1.52) and no-nausea endpoints (RR = 1.41; 95% CI, 1.18–1.68) (Chiu et al., 2016).

In contrary to updated guideline recommendation, olanzapine was not administered for any of 39 patients who had received highly emetogenic potential regimens in our institue. Our clinicians were reluctant to prescribe oral prophylaxis drugs in hospitalized patients.On the other hand, there was a fear of prescribing antipsychotic medications regarding the adverse effects.The main possible reason would be the lack of knowledge for new updates in the latest version of ASCO clinical recommendations (Koth and Kolesar, 2017). Not prescribing olanzapine in prophylaxis regimen of CINV justifies the high percentage of non-adherence to ASCO clinical recommendations (Koth and Kolesar, 2017). 

Firstly, the phenothiazine family was demonstrated to provide significant control over CINV, and high-dose metoclopramide was effectively used in preventing CINV even in patients receiving highly emetogenic potential regimens. By introducing 5-hydroxytryptamine type 3 (5-HT3) receptor antagonists, such as ondansetron, with better efficacy and toxicity profile, the use of metoclopramide for CINV prophylaxis was supplanted.

Comparison between the CINV prophylaxis’s effect of olanzapine with metoclopramide was evaluated in a trial conducted in 2013. Two hundred and seventy-six patients receiving highly emetogenic chemotherapy regimens were enrolled in this trial. Among these patients, 112 patients developed breakthrough nausea and vomiting while they were randomly assigned to received olanzapine 10 mg orally daily for 3 days or metoclopramide 10 mg orally TID for 3 days. All of their patients received initial prophylaxis with dexamethasone, palonosetron, and fosaprepitant. During the 72-hour observation period after breakthrough nausea and vomiting, patients who were treated with olanzapine were more likely than patients who were treated with metoclopramide to have no emesis (70% v 31%; P, 0.01) and no nausea (68% v 23%; P, 0.01). There were no grade 3 or 4 adverse effects. According to MD Anderson Symptom Inventory, scores for symptoms, such as sedation, did not differ significantly between the two study arms (Navari and Gray, 2013).

The unnecessary prescription of metoclopramide was repeatedly recorded in all four groups of chemotherapy according to emetogenic potential. The extra usage of unnecessary metoclopramide in the prevention of acute CINV by the majority of our physicians showed that they were not aware of exclusion of metoclopramide from guidelines or substitution of this medication with olanzapine.

In clinical practice, both patients and physicians are somewhat reluctant to hinder the use of antiemetics during follow-up visits, subsequently overdosed of CINV prophylaxis is likely to be maintained.

Noteworthy, there are negligible differences among different guidelines in how to categorize medications as the highly emetogenic or moderately emetogenic potential individually or in combination. For example, adriamycin or cyclophosphamide is categorized as moderately emetogenic agents separately, but when administered concomitantly (AC regimen), this combination is classified as highly emetogenic agents in NCCN guidelines (Tageja and Groninger, 2016). The recommendations of three important guidelines, namely ASCO, MASSC-ESMO, and NCCN concering prophylaxis of CINV in AC containing regimens and non- AC are different (Tageja and Groninger, 2016). The differences among guidelines’ recommendation could induce overestimation of non-adherence with guidelines in our study. However, the fundamental recommendations of all well-known guidelines (Tageja and Groninger, 2016) bear a resemblance to each other. 

The poor compatibility between medication prescribed for the management of CINV in our institute and ASCO guideline recommendations arises two important questions: how can we improve the current situation? and how can this nasty condition affect patients’ clinical outcome and financial burden on the public health system? 

Adherence to the guideline is related to better control of CINV. In a cohort study, O’Kane revealed that adhering to MASCC antiemetic guidelines significantly ameliorated CINV with cisplatin and oxaliplatin (O’Kane, 2009). 

Although some of investigators have evaluated antiemetic guideline outcomes with respect to cost containment (Nolte et al., 1998; Teich et al., 2000), few have assessed the effect of guideline implementation on patient care from the symptom-control perspective. Our center is a teaching hospital and most of the oncologists had previously underwent periodical education in supportive care. However, our study showed the need for continued learning. However, Mertens et al., (2003) demonstrated that training alone did not substantially improve adherence to published guideline recommendation.

Some strategies were suggested by other centers to reduce the guideline’s non-adherence rate of medications for prophylaxis of CINV. It has shown that pharmacy- or nursing-controlled prescriptions and software ordering tools can enhance guideline-based adherence (Nolte et al., 1998; Teich et al., 2000). Implementing the software-based prophylaxis of CINV and continuing educational programs were considered for our center to improve the guideline-based clinical practice and reduce overuse and potential harm in our patients.

In conclusion, although our center is a referral and university-affiliated center, the adherence to the ASCO 2017 (Hesketh et al., 2017) guideline recommendations for the prophylaxis of CINV at our institution was poor due to lack of prescribing olanzapine and over prescription of serotonin, neurokinin antagonists, and dopamine antagonists. Better communication and implementation of antiemetic guidelines must be considered as one practical way to reduce the burden of CINV.
